# A systems biology approach to understanding the regulation of lignin biosynthesis

**DOI:** 10.1186/1753-6561-5-S7-I14

**Published:** 2011-09-13

**Authors:** Vincent Chiang

**Affiliations:** 1 Forest Biotechnology Group, North Carolina State University, USA

## 

Lignin is a complex phenolic structural component of the secondary cell walls of all vascular plants. It is an irreversible end point of a major metabolic pathway in plant secondary metabolism. Lignin is fundamental to the adaptation of plants to land, the evolution of vascular transport and the resistance of plants to pests and pathogens. Lignin is a major barrier to the utilization of biomass for energy, for papermaking, and for forage digestibility due to the interaction of lignin with cellulosics in the plant cell secondary wall.

The past research on lignin biosynthesis is substantial, creating one of a few opportunities in higher plants to integrate genomics, proteomics, biochemistry, chemistry and modeling to develop a comprehensive understanding of biosynthesis and structure of a major component of morphology and adaptation. We are conducting a systems biology study on regulation of lignin biosynthesis in wood formation. We seek to build models to quantitatively illustrate how the entire pathway is organized and regulated and to reveal regulatory and metabolic flux control mechanisms, leading to lignin quantity and structures. We use the model woody plant,* Populus trichocarpa* (Nisqually-1), and the systems approach including advanced quantitative methods of genomics, proteomics, metabolomics, biochemistry and structural chemistry, to provide a comprehensive analysis of the regulation of lignin biosynthesis. A perturbation strategy is used to systematically knock down the expression of all pathway and regulatory genes known to be involved in lignin biosynthesis during wood formation, and the effects on lignin biosynthesis (gene transcripts, proteins, metabolites, quantity and structures) analyzed using advanced genomic methods available. This information forms the foundation of statistics-based mechanistic modeling and lignin quantity/structural predictions for a quantitative model of lignin biosynthesis. Details of systems data generation, data analyses and model development will be presented. Substantial data have been generated for gene-specific (amiRNA & RNAi) transgenic *P. trichocarpa*, enzyme kinetics, protein regulation and numerical modeling, and stable-isotope-dilution based absolute quantitation of proteins and of metabolites. These results will be discussed. Our long term goal is a predictive model of lignin biosynthesis and quantity/structure for greater understanding of the plant response to environmental stress and for more precise strategies to improve plant productivity and the production of energy, biomaterials and food.

